# Presence of Anxiety and Depression Symptoms Affects the First Time Treatment Efficacy and Recurrence of Benign Paroxysmal Positional Vertigo

**DOI:** 10.3389/fneur.2018.00178

**Published:** 2018-03-21

**Authors:** Wei Wei, Zahra N. Sayyid, Xiulan Ma, Tian Wang, Yaodong Dong

**Affiliations:** ^1^Department of Otology, Shengjing Hospital of China Medical University, Shenyang, China; ^2^Department of Otolaryngology-Head and Neck Surgery, Stanford University School of Medicine, Stanford, CA, United States; ^3^Department of Otolaryngology-Head and Neck Surgery, Second Xiangya Hospital of Central South University, Changsha, China

**Keywords:** psychiatric symptoms, anxiety, depression, benign paroxysmal positional vertigo, canalith repositioning maneuver, treatment efficacy, recurrence

## Abstract

**Objectives:**

To investigate the possible effects of anxiety and/or depression symptoms on the treatment outcomes and recurrence of benign paroxysmal positional vertigo (BPPV).

**Methods:**

This is a retrospective study conducted at a single institution. 142 consecutive patients diagnosed with idiopathic BPPV at the Department of Otology in Shengjing Hospital of China Medical University between October 2016 and July 2017 were retrospectively reviewed. 127 patients were finally included in this study. Zung self-rating anxiety scale (SAS) and Zung self-rating depression scale (SDS) were used to evaluate the presence of anxiety and/or depression, respectively, in our BPPV patients. A significant score (at or above 50 for SAS and 53 for SDS) represents the presence of clinically significant symptoms. Two-tailed Student’s *t*-test, χ^2^ test, and logistic regression analysis were used as appropriate. A *p* value less than 0.05 was considered statistically significant.

**Results:**

The prevalence of anxiety and/or depression symptoms in BPPV patients in the present study was 49.61%. The effectiveness of the first time canalith repositioning maneuver (CRM) was 70.08%. With weekly follow-up treatments of CRM, the success rate increased to 97.64% by 1 month. The total recurrence rate at 6-month follow-up post-cure was 14.17%. Holding all other variables constant, patients with psychiatric symptoms (Relative-risk ratio: 3.160; *p* = 0.027) and patients with non-posterior semicircular canal (PSC) involvement (Relative-risk ratio: 7.828, *p* = 0.013) were more likely to experience residual dizziness (RD) even after effective CRM treatment. Psychiatric symptoms (Relative-risk ratio: 6.543; *p* = 0.001) and female gender (Relative-risk ratio: 4.563; *p* = 0.010) are risk factors for the failure of first time CRM. In addition, BPPV patients with psychiatric symptoms (Odds ratio: 9.184, *p* = 0.008) were significantly more likely to experience recurrences within the first 6 months after a successful maneuver.

**Conclusion:**

Anxiety-depression status significantly reduced the efficacy of the first time CRM and increased the risk for recurrence. Other factors, such as female gender and non-PSC involvement are also susceptible risk factors for BPPV patients to require multiple treatments and experience delayed recovery. A screening for psychiatric symptoms in BPPV patients and active treatment of these symptoms would benefit both physicians and patients in understanding and improving the prognosis of the disease and treatment options.

## Introduction

Benign paroxysmal positional vertigo (BPPV) is an idiopathic vestibular disease that manifests with transient paroxysmal vertigo and nystagmus stimulated by changes in head position. The lifetime prevalence of BPPV is around 2.4% (1). BPPV is the most common vestibular disorder and accounts for 17–42% of all dizziness complaints in the United States (2). In the Chinese population, 36.5% of patients with peripheral vertigo ultimately receive a diagnosis of BPPV (3). Given the noteworthy prevalence, BPPV represents a large burden on the health care system and its patients.

The mechanism of BPPV is an inappropriate stimulation of the semicircular canal ampullae that is induced by free-floating otoconia from the utricle (4). The onset of BPPV can be associated with head trauma, a prolonged recumbent position, and various pathological processes of the inner ear. Systemic diseases including hypertension, diabetes, hypercholesterolemia, cerebrovascular ischemia, and cervical spondylosis may also exacerbate the degeneration of the posterior labyrinth, facilitating the detachment of otoconia (5, 6), which in turn results in BPPV. Moreover, an increased risk for BPPV was reported to be associated with migraine in a population-based study, which may be ascribed to the repetitive disturbance of vestibulocochlear microvasculature resulting in damage of the vestibular epithelium (7). The main treatment method for BPPV is performing a canalith repositioning maneuver (CRM), which aims to relocate the dislodged otoconia back into the vestibule. Although more than 95% of BPPV cases can be resolved by using CRM (8), the treatment efficacy on first attempt differs from person to person. A small portion of cases is not resolved with first time CRM (failed CRM), as patients continue to experience persistent vertigo and nystagmus post-maneuver. These patients receive increased maneuver attempts and experience significantly delayed recovery. Unfortunately, 31–61% of patients with a successful CRM (due to the absence of positional vertigo and nystagmus) experience feeling of prolonged imbalance, or residual dizziness (RD) (9–11). To resolve this, these patients also require increased maneuver attempts even though the CRM was determined to be clinically successful. There is also a noticeably high recurrence rate in certain populations of BPPV patients such as those with multiple comorbidities (6, 12). However, the mechanisms underlying the differences in first time CRM efficacy and BPPV recurrence are still unclear.

A growing body of evidence supports the notion that psychological factors can influence disease activity and response to therapy in a complex manner (13–15). The coexistence of vestibular diseases and psychiatric disorders has been repeatedly described in the literature (16–21). In particular, psychological factors may influence recovery from balance disorders, resulting in a prolonged vertigo or dizziness (22–24). However, to our knowledge, no study to date has focused on addressing how these psychological factors may affect the treatment efficacy or recurrence rate of BPPV. Therefore, investigating the possible effects of psychological factors on treatment outcome and BPPV recurrence will be of great significance to gain better insights into improving short- and long-term treatment efficacy for patients with BPPV.

In this study, two psychometric scales—Zung self-rating anxiety scale (SAS) and Zung self-rating depression scale (SDS)—were used to screen patients for symptoms of anxiety and depression. We found that these two common psychiatric symptoms served as risk factors for an unsatisfactory outcome (failure or success with RD) of first time CRM. Consequently, patients with anxiety and/or depression symptoms required more treatment visits and experienced increased recurrence rates of BPPV than those without these psychiatric symptoms.

## Materials and Methods

### Ethical Consideration

This study has been approved by the Ethics Committee of Shengjing Hospital of China Medical University (protocol number 2017PS39K).

### Patients

One hundred and forty-two consecutive patients solely diagnosed with idiopathic BPPV at the department of Otology in Shengjing Hospital of China Medical University between October 2016 and July 2017 were retrospectively reviewed (patients comorbid with other types of peripheral vertigo including Ménière’s disease, vestibular neuritis and vestibular migraine were not reviewed). These 142 patients ranged in age from 14 to 78 years. We then excluded 15 patients who had the following conditions: (1) positive history of drug or alcohol abuse (*n* = 2); (2) prior diagnosis of posttraumatic stress disorder (*n* = 1); (3) diagnosis of BPPV and treatment with repositioning maneuver prior to being referred to our institution (*n* = 4); (4) incomplete medical record due to drop-out prior to successful maneuver or failure to appear at all follow-up visits (*n* = 5); and (5) diagnosis of central vertigo (*n* = 3).

### Diagnosis, Treatment, and Follow-Up

All patients received a complete battery of vestibular and audiological tests. The diagnosis of idiopathic BPPV was made according to the medical history and subjective symptoms together with objective signs of positional nystagmus identified during the Dix–Hallpike Test and/or the Supine Rolling Test. Comorbidities of systemic diseases were obtained *via* a detailed interrogation combined with the medical history recorded in the electronic medical record system and/or patients’ previous laboratory and imaging test results from our or other institutions.

Patients were treated using the appropriate canalith repositioning maneuver (CRM) according to the involved semicircular canal. Epley’s maneuver was performed for anterior semicircular canal (ASC) BPPV and posterior semicircular canal (PSC) BPPV, and the Barbecue maneuver was performed for lateral semicircular canal (LSC) BPPV. All patients returned to the clinic 1 week after receiving CRM to determine if successful treatment was achieved. Repositioning maneuver success was defined as reported relief of vertigo and nystagmus symptoms on positional testing (25). Patients who showed no nystagmus on positional testing but still expressed RD or unsteadiness were retreated with the same maneuvers until dizziness symptoms were fully resolved.

Once the CRM was determined as completely satisfactory (complete remission of vertigo and nystagmus with no RD present), patients were followed up monthly. They were also instructed to return to the clinic as soon as possible if they experienced any symptoms of recurrences throughout the follow-up period. Recurrence was defined as a confirmed relapse of vertigo and nystagmus according to the Dix–Hallpike Test or Supine Rolling Test after a successful treatment (26).

### Psychometric Instruments

Psychiatric assessments are not routinely evaluated in general dizziness patients presenting to neurotology clinics. In this study, Zung SAS (27) and Zung SDS (28) were used to measure the status of anxiety and depression in patients with BPPV. Both scales are reliable and have been validated among Chinese populations (29). The scoring procedure was performed as previously described with cutoff scores at 50 and 53 for SAS and SDS, respectively (21). A significant score (at or above the cutoff) resulted in a positive assessment of either anxiety and/or depression status. Psychometric questionnaires were completed prior to receiving CRM.

### Statistical Analysis

Stata software (Version 13.0, StataCorp) was used for statistical analysis. Two-tailed Student’s *t*-test (Table 1. Average age), χ^2^ test (Tables 1 and 2.), and multivariate logistic regression analysis (Tables 3 and 4) were used as appropriate. A *p* value less than 0.05 was considered statistically significant.

**Table 1:** Demographic information on BPPV patients.

Demographic data	Total		psy-BPPV		i-BPPV		*p* Value
	*N*	%	*N*	%	*N*	%	
*N*	127	100	63	49.61	64	50.39	N/A
Age (average years)^a^	53.90 ± 13.93		59.27 ± 9.73		48.61 ± 15.42		0.000***
<60	78	65.42	30	47.62	48	75.00	0.002**
≥60^b^	49	38.58	33	52.38	16	25.00	
**Gender^b^**							
Male	46	36.22	19	30.16	27	42.19	0.158
Female	81	63.78	44	69.84	37	57.81	
**Laterality^b^**							
Right	80	62.99	41	65.08	39	60.94	0.629
Left	47	37.01	22	34.92	25	39.06	
**Involved semicircular canal^b^**							
PSC	107	84.25	55	87.30	52	81.25	0.349
n-PSC	20	15.75	8	12.70	12	18.75	
Comorbidity^b^	84	66.14	48	76.19	36	56.25	0.018*
Hypertension	37	29.13	23	36.51	14	21.88	0.070
Diabetes	19	14.96	12	19.05	7	10.94	0.200
Hypercholesterolemia	13	10.24	7	11.11	6	9.38	0.747
Migraine	9	7.09	7	11.11	2	3.12	0.079
Cerebrovascular ischemia	35	27.56	23	36.51	12	18.75	0.025*
Cervical spondylosis	57	44.88	34	53.97	23	35.94	0.041*

*^a^Mean ± SD, two-tailed Student’s t-test was used to compare differences between the two groups*.

*^b^χ^2^ test was used to compare differences between the two groups*.

*p < 0.05.

**p < 0.01.

***p < 0.001.

*i-BPPV, idiopathic Benign Paroxysmal Positional Vertigo; psy-BPPV, i-BPPV with anxiety and/or depression symptoms; PSC, posterior semicircular canal BPPV; n-PSC, non-posterior semicircular canal BPPV (lateral semicircular canal and anterior semicircular canal)*.

**Table 2:** Treatment outcomes of BPPV patients.

Follow-up exam		Total		psy-BPPV		i-BPPV		*p* Value
		*N*	%	*N*	%	*N*	%	
**1-week^a^**								
Single	Success w/o RD	41	32.28	12	19.05	29	45.31	0.000***
Multiple	Success with RD	48	37.80	23	36.51	25	39.06	
	Failure	38	29.92	28	44.44	10	15.63	
**1-month^a^**								
Success w/o RD		111	87.40	53	84.13	58	90.63	0.537
Success with RD		13	10.24	8	12.70	5	7.81	
Failure		3	2.36	2	3.17	1	1.56	
**3-month**								
Success w/o RD		127	100%	63	100%	64	100%	N/A
**Recurrence after complete resolution^a^**								
3 months		16	12.60	14	22.22	2	3.12	0.001**
6 months		18	14.17	16	25.40	2	3.12	0.000***

*^a^χ^2^ test was used to compare difference (success or failure) between the two groups*.

*RD, residual dizziness*.

**p < 0.01.

***p < 0.001.

**Table 3:** Multinomial logistical regression analysis of one-week treatment outcome of BPPV.

Parameter	*p* Value	Relative-risk ratio (95% CI)
**Residual dizziness**		
Age (≥60)	0.841	0.901 (0.325–2.499)
Gender (female)	0.066	2.514 (0.940–6.724)
Laterality	0.970	1.019 (0.386–2.688)
Involved semicircular canal (n-PSC)	0.013*	7.828 (1.551–39.508)
Psychiatric disorders	0.027*	3.160 (1.139–8.769)
Comorbidity		
Hypertension	0.710	0.796 (0.239–2.646)
Diabetes	0.995	1.005 (0.225–4.483)
Hypercholesterolemia	0.450	1.918 (0.354–10.377)
Migraine	0.062	0.903 (0.072–1.131)
Cerebrovascular ischemia	0.778	1.198 (0.342–4.192)
Cervical spondylosis	0.405	0.658 (0.246–1.763)
**Failure**		
Age (≥60)	0.974	1.019 (0.334–3.105)
Gender (female)	0.010*	4.563 (1.431–14.547)
Laterality	0.856	0.904 (0.304–2.686)
Involved semicircular canal (n-PSC)	0.091	4.718 (0.782–28.445)
Psychiatric disorders	0.001**	6.543 (2.110–20.289)
Comorbidity		
Hypertension	0.829	1.151 (0.322–4.112)
Diabetes	0.530	1.640 (0.350–7.677)
Hypercholesterolemia	0.527	1.785 (0.296–10.764)
Migraine	0.790	0.782 (0.128–4.779)
Cerebrovascular ischemia	0.124	2.630 (0.766–9.023)
Cervical spondylosis	0.957	1.030 (0.345–3.076)

*p < 0.05.

**p < 0.01.

*BPPV, benign paroxysmal positional vertigo; n-PSC, non-posterior semicircular canal (lateral semicircular canal and anterior semicircular canal); RD, residual dizziness*.

## Results

**Figure 1:**
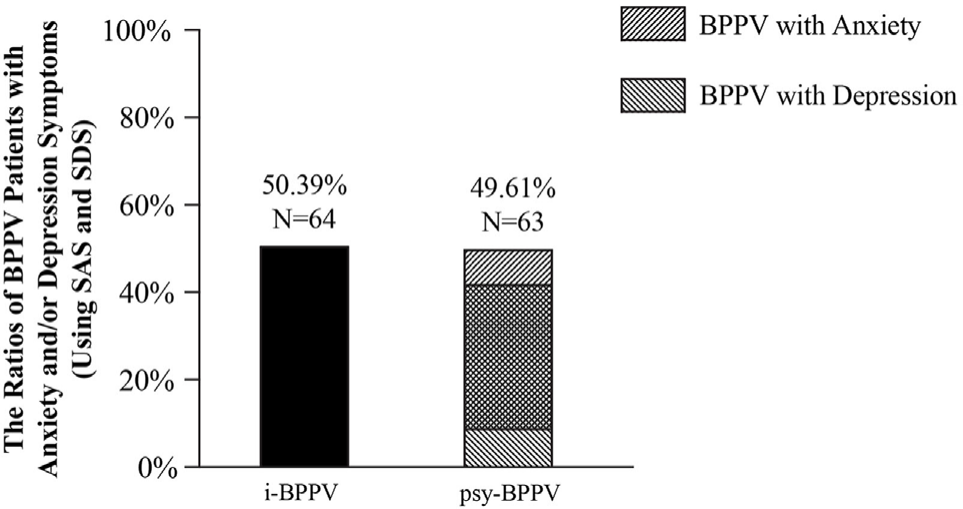
The ratios of anxiety and/or depression symptoms in BPPV patients.

Among all 127 patients with idiopathic BPPV, 52 (40.94%) patients were found to have clinically significant anxiety symptoms and 53 (41.73%) patients were considered to have clinically significant depression symptoms. 42 (33.07%) patients were found to have both. Patients with anxiety and/or depression symptoms were grouped into the psychiatric symptoms group (psy-BPPV, *N* = 63, 49.61%), and those with insignificant scores on both questionnaires were grouped into the idiopathic BPPV group (i-BPPV, *N* = 64, 50.39%) (Figure 1). The psy-BPPV group had a significantly higher proportion of patients 60 years and older compared to the i-BPPV group (52.38% versus 25.00%, *p* = 0.002) (Table 1). There was no significant difference in gender ratio (female/male patients, *p* = 0.158) and in laterality of affected ear (right/left ear, *p* = 0.629). The PSC was most frequently involved, with 107 (84.25%) patients diagnosed in total. Patients diagnosed with LSC- or ASC-BPPV were grouped into the n-PSC BPPV group. There was no significant difference in the ratio of involved canal (n-PSC/PSC) between the two groups (*p* = 0.349). Patients in the psy-BPPV group had significantly more total comorbidities than the i-BPPV group (74.60% versus 54.69%, *p* = 0.019). This difference is likely attributed to the increased prevalence of both cerebrovascular disease and cervical spondylosis (*p* = 0.025 and *p* = 0.041, respectively) in the psy-BPPV group. However, a causal relationship cannot be drawn between psychiatric symptoms and those two comorbidities due to the unmatched distribution of patient age in the two groups.

Patients were treated with the Epley or Barbecue maneuver depending on the involved semicircular canal. Treatment outcomes are listed in Table 2. In total, 89 patients (70.08%) were successfully treated with a single maneuver treatment, but the success rate of first time CRM was significantly lower in the psy-BPPV group than the i-BPPV group (55.56% versus 84.37%, *p* < 0.001). Among these successfully treated patients, 48 (37.80%) reported for a feeling of prolonged unsteadiness or dizziness (RD) during the first week of follow-up. Patients with RD and patients with failed first attempt CRM were subsequently treated once a week until satisfactorily cured. The cumulative success rate increased to 124 patients (97.64%) at 1 month with no significant difference between the two groups by 1 month (96.83% psy-BPPV versus 98.44% i-BPPV, *p* = 0.537). The total success rate within 5 weeks of treatment was 100% for both groups. This finding suggests that psychological symptoms may only have a significant effect on the efficacy of first time CRM, but not necessarily on multiple treatments.

Next, we sought to explore if psychiatric symptoms influence recurrence rates of BPPV. Once a satisfactory treatment was attained, patients were followed up monthly. By the 3-month follow-up visit, 16 (12.60%) patients were diagnosed with recurrent BPPV. Significantly more patients in the psy-BPPV group experienced recurrence of BPPV compared to the i-BPPV group (22.22% versus 3.12%, *p* = 0.001). The cumulative recurrence rate increased to 14.17% by 6 months. A significantly higher recurrence rate was again observed in the psy-BPPV group compared to the i-BPPV group (25.40% versus 3.12%, *p* < 0.001).

The outcome and/or recurrence of BPPV could also be adversely affected by increased age and the presence of comorbidities. Using multinomial logistic regression analysis to hold all other variables constant, we found that the presence of psychiatric symptoms, gender, and involved semicircular canal are independent factors that significantly affect the first time treatment efficacy of BPPV. Patients with clinically significant anxiety and/or depression symptoms (Relative-risk ratio: 3.160, *p* = 0.027) and patients with non-PSC involvement (Relative-risk ratio: 7.828, *p* = 0.013) have higher risk to experience RD even after receiving an effective CRM treatment. Anxiety-depression status (Relative-risk ratio: 6.543, *p* = 0.001) and female gender (Relative-risk ratio: 4.653, *p* = 0.010) serve as risk factors for the failure of CRM treatment (Table 3). In addition, the presence of anxiety and/or depression symptoms (Odds ratio: 9.184, *p* = 0.008) also significantly increased the risk of BPPV recurrence during the first 6 months after a satisfactory cure (Table 4). These results further confirmed our previous findings that psychiatric symptoms adversely affect the treatment efficacy of first time CRM and recurrence of BPPV.

**Table 4:** Logistical regression analysis of recurrence of BPPV within 6 months.

Parameter	*p* Value	Odds ratio (95% CI)
Age (≥60)	0.744	1.248 (0.331–4.702)
Gender (female)	0.818	0.863 (0.246–3.029)
Laterality	0.887	1.094 (0.315–3.807)
Involved semicircular canal (n-PSC)	0.325	2.155 (0.468–9.933)
Psychiatric disorders	0.008**	9.184 (1.802–46.801)
Comorbidity		
Hypertension	0.853	0.882 (0.235–3.316)
Diabetes	0.254	2.442 (0.527–11.310)
Hypercholesterolemia	0.193	2.967 (0.577–15.253)
Migraine	0.864	1.170 (0.194–7.074)
Cerebrovascular ischemia	0.260	2.036 (0.591–7.011)
Cervical spondylosis	0.400	1.712 (0.489–5.987)

**p < 0.01.

*BPPV, benign paroxysmal positional vertigo; n-PSC, non-posterior semicircular canal (lateral semicircular canal and anterior semicircular canal)*.

## Discussion

### Synopsis of Key Findings

Studies conducted over the past few decades have suggested that psychological problems are associated with vertigo and balance problems. Whereas vestibular dysfunction can engender psychiatric disorders (16, 23, 30), anxiety or depressive disorders may also serve as the primary cause of vestibular symptoms (31). This results in the formation of a vicious cycle, which is likely to exacerbate both psychiatric and vestibular disorders. However, few studies to date have put emphasis on whether and how this unhealthy psychological status might affect the efficacy of treatments for vestibular dysfunction. Here, by focusing on the most common vestibular disorder (BPPV), we found that the presence of anxiety and/or depression symptoms adversely affects the efficacy of first time CRM by increasing the risk of patients experiencing RD or receiving failed treatment. It also served as risk factors for BPPV recurrence within 6 months post-cure.

In our study, 37.80% of patients with a clinically defined successful CRM received follow-up CRM treatments due to the presence of RD. RD in BPPV patients after successful CRM could be a physiological sensation due to the presence of residual debris within the semicircular canals which are insufficient to provoke nystagmus (32). However, RD may also appear as a particular form of psychogenic dizziness. Anxiety disorders account for 8–10% of cases with vestibular symptoms as chief complaints (31). Two common manifestations of anxiety disorders are generalized anxiety disorder and panic attacks. Patients with generalized anxiety disorder can frequently experience dizziness, lightheadedness, and unsteadiness (33). Recurrent episodes of BPPV have the propensity to trigger chronic worry and results in exacerbation of the psychosomatic symptoms of anxiety. Panic attacks—acute episodes of physical and psychological symptoms that may occur in response to fear conditions—may also serve as a psychiatric cause of vestibular symptoms (33). The most common manifestations of panic attacks are cardiopulmonary symptoms (chest pain, palpitations, and dyspnea) and vestibular symptoms (non-vertiginous dizziness, lightheadedness, and unsteadiness) (34). Acute and episodic vertigo can also trigger panic attacks due to fear of falling, especially when comorbid with anxiety. When patients experience numerous panic attacks concomitant with a debilitating fear of future attacks, this disorder is considered a panic disorder. Common symptoms of panic disorder include dizziness and unsteadiness as well. In addition, up to 20% of patients with anxiety experience phobic postural vertigo (PPV), a functional vestibular syndrome including lightheadedness and varying degrees of unsteadiness that is often secondary to organic vestibular disease (35, 36). This may offer another possible explanation for the generation of RD.

The aim of the reposition maneuver for BPPV is to relocate dislodged otoliths back into the vestibule and allow for their reabsorption by vestibular dark cells. Thus far, no established pathophysiological link exists between psychiatric disorders and failure of initial repositioning maneuver or increased risk of BPPV recurrence. Several previous studies have revealed that the otolith dysfunction in BPPV patients may be partly due to a degenerative process (37–39) that correlates with oxidative stress and pro-inflammatory responses (40). Tsai and colleagues recently observed high levels of pro-inflammatory cytokines and oxidative stress products in patients’ plasma during BPPV attacks (41). These pro-inflammatory responses were corrected and the oxidative stress was attenuated after a successful repositioning maneuver. Numerous epigenetic modulators like SIRT1, Cox2, FoxO, and P53 play crucial roles during this degenerative and recovery process, and maintaining a subtle balance among these modulators, reactive oxygen species, and pro-inflammatory cytokines is necessary for the complete recovery of BPPV (41). Interestingly, an extensive body of literature has shown that both anxiety and depression are also associated with a chronic low-grade inflammatory response (42, 43), increased oxidative and nitrosative stress, and activation of cell-mediated immunity (44, 45). This may further exacerbate the vestibular degeneration, consequently leading to difficulty with the relocation and reabsorption of the dislodged otoliths. Moreover, the persistence of anxiety/depression may disrupt the balance of epigenetic modulation after a successful maneuver, resulting in a higher recurrence rate within a short period of time. Both anxiety and depression have also been linked to neuroendocrine dysfunction (46, 47), which may imbalance the blood flow to the inner ear, thus influencing the recovery of BPPV (48).

### Comparisons With Other Studies

The main characteristics of our patient population were comparable with those published in other relevant studies. For example, previous studies reported that the most common age of onset for BPPV is around 50 (1), and the number of women affected was 1.5-fold more than that of men (49). In our study, the mean age of patients was 53.90 ± 13.93 and the ratio of females to males was 1.76 (Table 1). This gender discrepancy may be due to menopause-related hormonal fluctuations: a large retrospective study suggested that low estrogen levels might account for the high prevalence of BPPV in perimenopausal and older women (50). Another study posited that estrogen deficiency might influence calcium metabolism, thus resulting in degeneration of otoconia (51). These mechanisms may also partly address why female BPPV patients are less likely to be cured on first attempt of the repositioning maneuver, thus requiring more treatment visits.

We also observed a difference in laterality in our study, with the right labyrinth more predominantly affected (Table 1). Similar results have been published in a meta-analysis conducted by von Brevern et al. (52). The PSC served as the most frequently involved semicircular canal in our study group (Table 1), and those with ASC or LSC involvement received more treatments visits, consistent with previous findings (53).

Although the efficacy of first time CRM was significantly affected by psychiatric symptoms (Tables 2 and 3), the total cumulative success rate at 1 month was 97.64%, which is slightly higher than the previously reported range of 70–95% (54, 55). Comorbidities, such as hypertension and diabetes, had no significant effect on the treatment outcome of CRM, again consistent with previous findings (54, 56). Lastly, it has been reported that BPPV patients who received higher numbers of maneuvers tended to have the greatest risk of recurrence, and the relapse largely occurs within the first few months (12). In our study, patients in the psy-BPPV group received increased maneuver attempts and had a significantly higher recurrence rate (Tables 2 and 4), which is perfectly in accordance with previous reports. However, a causal relationship between the increased maneuver attempts and the recurrence of BPPV cannot be determined from our study. Nevertheless, we did not detect a correlation between the presence of comorbidities and increased recurrence rates of BPPV, as reported previously (6, 12). This may be due to the length of the follow-up period in this study, which may not have been long enough to identify all recurrence events.

### Strengths and Limitations of the Study

As discussed previously, psychiatric factors may affect both the onset and the recovery of BPPV. Few studies have examined psychiatric symptoms as factors that may affect treatment outcomes of BPPV and as precursors of its recurrence. Staab et al. reported a single case of a 41-year-old woman with several combined neurotologic diseases as well as health anxiety, and suggested that psychological factors could have adversely affected her treatment outcomes (57). Results from our study support this assessment. The clinical characteristics of our patient population are also fully comparable to other studies. Collectively, results from the previous reports mentioned above along with our representative patient demographics strongly reinforce the dependability of our study.

There are, however, a few limitations of this study. First, a causal relationship cannot be drawn between psychiatric symptoms and BPPV due to the retrospective study design. Whether the psychiatric symptoms triggered the BPPV attack or the repeated attack of BPPV generated the secondary emotional dysfunction cannot be determined. Also, the estimation of psychological status was not repeated prior to assessment of BPPV clearance. Therefore, we were not able to assess the anxiety linked to the first medical visit. Future studies designed in a prospective manner can help address these questions. Second, we did not find any significant correlations between comorbidities of systemic diseases and either treatment outcome or recurrence rates. However, the prevalence of comorbidities in our patient population was likely an underestimate, since they were obtained *via* historical records from patients’ medical charts and/or test results. It is likely that some patients did not receive regular health check-ups and/or avoided seeking medical treatment due to the modest health burdens of their systemic disease, ultimately leading to an underreporting of comorbidity status. Comorbidities may thus play some role in effecting treatment outcome and/or recurrence rates of BPPV that is indiscernible in our study. Third, because recurrence was only tracked through the first 6 months after cure, relapses that occurred after that the 6-month time point would not have been documented. However, since a significant association between psychiatric disorders and recurrence of BPPV was already identified, this would likely also underestimate the link between psychiatric disorders and BPPV recurrence.

## Conclusion

The presence of anxiety and/or depression symptoms significantly reduced the efficacy of the first time repositioning maneuver and increased the risk for recurrence. Female gender and non-PSC involvement are also risk factors for patients with BPPV to receive multiple treatments and delayed cure. Awareness of these results will allow us to better evaluate the prognosis of and improve treatment efficacy for patients with BPPV. Therefore, we recommend a screening for anxiety and depression symptoms in all patients coming to the clinic for BPPV assessment or treatment. Self-management of these psychiatric symptoms and/or psychiatric therapy could be helpful for improving the treatment efficacy and reducing the recurrence risk of BPPV.

## Author Contributions

YD conceived and led the study. WW, XM, and YD reviewed the patients’ medical record and acquired the data. TW and YD performed the statistical analysis of the data. WW, ZS, and YD wrote the manuscript with input from all co-authors.

## Conflict of Interest Statement

All authors declare that the research was conducted in the absence of any commercial or financial relationships that could be construed as a potential conflict of interest.

## References

[1] von Brevern M Radtke A Lezius F Feldmann M Ziese T Lempert T Epidemiology of benign paroxysmal positional vertigo: a population based study. J Neurol Neurosurg Psychiatry (2007) 78(7):710–5. 10.1136/jnnp.2006.100420 17135456 PMC2117684

[2] Bhattacharyya N Baugh RF Orvidas L Barrs D Bronston LJ Cass S Clinical practice guideline: benign paroxysmal positional vertigo. Otolaryngol Head Neck Surg (2008) 139(5 Suppl 4):S47–81. 10.1016/j.otohns.2008.08.022 18973840

[3] Dizziness Diagnostic Process Recommendation Expert Group. Guideline for the diagnosis of dizziness (in Chinese). Chin J Int Med (2009) 48(5):435–7. 10.3760/cma.j.issn.0578-1426.2009.05.030

[4] Fife TD Iverson DJ Lempert T Furman JM Baloh RW Tusa RJ Practice parameter: therapies for benign paroxysmal positional vertigo (an evidence-based review): report of the quality standards subcommittee of the American Academy of Neurology. Neurology (2008) 70(22):2067–74. 10.1212/01.wnl.0000313378.77444.ac 18505980

[5] Zhang Y Chen X Wang X Cao L Dong Z Zhen J A clinical epidemiological study in 187 patients with vertigo. Cell Biochem Biophys (2011) 59(2):109–12. 10.1007/s12013-010-9120-1 20976571

[6] De Stefano A Dispenza F Suarez H Perez-Fernandez N Manrique-Huarte R Ban JH A multicenter observational study on the role of comorbidities in the recurrent episodes of benign paroxysmal positional vertigo. Auris Nasus Larynx (2014) 41(1):31–6. 10.1016/j.anl.2013.07.007 23932347

[7] Chu C-H Liu C-J Lin L-Y Chen T-J Wang S-J. Migraine is associated with an increased risk for benign paroxysmal positional vertigo: a nationwide population-based study. J Headache Pain (2015) 16(1):62. 10.1186/s10194-015-0547-z 26141381 PMC4491067

[8] Dorigueto RS Mazzetti KR Gabilan YP Gananca FF. Benign paroxysmal positional vertigo recurrence and persistence. Braz J Otorhinolaryngol (2009) 75(4):565–72. 10.1016/S1808-8694(15)30497-3 19784427 PMC9446034

[9] Kim H-A Lee H. Autonomic dysfunction as a possible cause of residual dizziness after successful treatment in benign paroxysmal positional vertigo. Neurophysiol Clin (2014) 125(3):608–14. 10.1016/j.clinph.2013.08.008 24045026

[10] Seok JI Lee HM Yoo JH Lee DK. Residual dizziness after successful repositioning treatment in patients with benign paroxysmal positional vertigo. J Clin Neurol (2008) 4(3):107–10. 10.3988/jcn.2008.4.3.107 19513312 PMC2686873

[11] Teggi R Quaglieri S Gatti O Benazzo M Bussi M. Residual dizziness after successful repositioning maneuvers for idiopathic benign paroxysmal positional vertigo. ORL J Otorhinolaryngol Relat Spec (2013) 75(2):74–81. 10.1159/000350255 23774304

[12] Perez P Franco V Cuesta P Aldama P Alvarez MJ Mendez JC. Recurrence of benign paroxysmal positional vertigo. Otol Neurotol (2012) 33(3):437–43. 10.1097/MAO.0b013e3182487f78 22388730

[13] Andrews H Barczak P Allan RN. Psychiatric illness in patients with inflammatory bowel disease. Gut (1987) 28(12):1600–4. 10.1136/gut.28.12.1600 3428687 PMC1433950

[14] Musselman DL Evans DL Nemeroff CB. The relationship of depression to cardiovascular disease: epidemiology, biology, and treatment. Arch Gen Psychiatry (1998) 55(7):580–92. 10.1001/archpsyc.55.7.580 9672048

[15] Persoons P Vermeire S Demyttenaere K Fischler B Vandenberghe J Van Oudenhove L The impact of major depressive disorder on the short- and long-term outcome of Crohn’s disease treatment with infliximab. Aliment Pharmacol Ther (2005) 22(2):101–10. 10.1111/j.1365-2036.2005.02535.x 16011668

[16] Eckhardt-Henn A Dieterich M Psychiatric disorders in otoneurology patients. Neurol Clin (2005) 23(3):731–49, vi. 10.1016/j.ncl.2005.01.008 16026674

[17] Furman JM Redfern MS Jacob RG. Vestibulo-ocular function in anxiety disorders. J Vestib Res (2006) 16(4–5):209–15. 17538210

[18] Jacob RG Furman JM. Psychiatric consequences of vestibular dysfunction. Curr Opin Neurol (2001) 14(1):41–6. 10.1097/00019052-200102000-00007 11176216

[19] Staab JP Ruckenstein MJ. Which comes first? Psychogenic dizziness versus otogenic anxiety. Laryngoscope (2003) 113(10):1714–8. 10.1097/00005537-200310000-00010 14520095

[20] Staab JP Ruckenstein MJ. Chronic dizziness and anxiety: effect of course of illness on treatment outcome. Arch Otolaryngol Head Neck Surg (2005) 131(8):675–9. 10.1001/archotol.131.8.675 16103297

[21] Yuan Q Yu L Shi D Ke X Zhang H Anxiety and depression among patients with different types of vestibular peripheral vertigo. Medicine (2015) 94(5):e453 10.1097/MD.0000000000000453 25654382 PMC4602710

[22] Yardley L Redfern MS. Psychological factors influencing recovery from balance disorders. J Anxiety Disord (2001) 15(1–2):107–19. 10.1016/S0887-6185(00)00045-1 11388354

[23] Godemann F Koffroth C Neu P Heuser I. Why does vertigo become chronic after neuropathia vestibularis? Psychosom Med (2004) 66(5):783–7. 10.1097/01.psy.0000140004.06247.c9 15385707

[24] Godemann F Siefert K Hantschke-Brüggemann M Neu P Seidl R Ströhle A. What accounts for vertigo one year after neuritis vestibularis-anxiety or a dysfunctional vestibular organ? J Psychiatr Res (2005) 39(5):529–34. 10.1016/j.jpsychires.2004.12.006 15992562

[25] Tan J Yu D Feng Y Song Q You J Shi H Comparative study of the efficacy of the canalith repositioning procedure versus the vertigo treatment and rehabilitation chair. Acta Otolaryngol (2014) 134(7):704–8. 10.3109/00016489.2014.899711 24807849

[26] Yamanaka T Shirota S Sawai Y Murai T Fujita N Hosoi H. Osteoporosis as a risk factor for the recurrence of benign paroxysmal positional vertigo. Laryngoscope (2013) 123(11):2813–6. 10.1002/lary.24099 23568754

[27] Zung WW A rating instrument for anxiety disorders. Psychosomatics (1971) 12(6):371–9. 10.1016/S0033-3182(71)71479-0 5172928

[28] Zung WW Richards CB Short MJ Self-rating depression scale in an outpatient clinic: further validation of the SDS. Arch Gen Psychiatry (1965) 13(6):508–15. 10.1001/archpsyc.1965.01730060026004 4378854

[29] Peng H Zhang Y Ji Y Tang W Li Q Yan X Analysis of reliability and validity of Chinese version of SDS scale in women of rural area. Shanghai Med Pharm J (2013) 14:20–2.

[30] Furman JM Balaban CD Jacob RG Marcus DA Migraine-anxiety related dizziness (MARD): a new disorder? J Neurol Neurosurg Psychiatry (2005) 76(1):1–8. 10.1136/jnnp.2004.048926 15607984 PMC1739317

[31] Staab JP Behavioural neuro-otology. In: Bronstein A, editor. Oxford textbook of vertigo and imbalance. Oxford, UK: Oxford University Press (2013). p. 333–46.

[32] Di GS Ottaviani F Scarano E Picciotti P Di NW. Postural control in horizontal benign paroxysmal positional vertigo. Eur Arch Otorhinolaryngol (2000) 257(7):372–5. 10.1007/s004050000243 11052247

[33] American Psychiatric Association. Diagnostic and Statistical Manual of Mental Disorders, 5th ed. Washington, DC: American Psychiatric Press (2013).

[34] Sklare DA Stein MB Pikus AM Uhde TW Disequilibrium and audiovestibular function in panic disorder: symptom profiles and test findings – biological psychiatry. Biol Psychiatry (1990) 25(7):A185 10.1016/0006-3223(89)91861-1 2240177

[35] Brandt T Phobic postural vertigo. Neurology (1996) 46(6):1515–9. 10.1212/WNL.46.6.1515 8649539

[36] Brandt T Dieterich M Strupp M Vertigo and Dizziness: Common Complaints. London: Springer-Verlag (2014). 10.1007/978-0-85729-591-0

[37] Fyrmpas G Barkoulas E Haidich AB Tsalighopoulos M. Vertigo during the Epley maneuver and success rate in patients with BPPV. Eur Arch Otorhinolaryngol (2013) 270(10):2621–5. 10.1007/s00405-012-2292-0 23203243

[38] Hong SM Park DC Yeo SG Cha CI. Vestibular evoked myogenic potentials in patients with benign paroxysmal positional vertigo involving each semicircular canal. Am J Otolaryngol (2008) 29(3):184–7. 10.1016/j.amjoto.2007.07.004 18439953

[39] von Brevern M Schmidt T Schonfeld U Lempert T Clarke AH. Utricular dysfunction in patients with benign paroxysmal positional vertigo. Otol Neurotol (2006) 27(1):92–6. 10.1097/01.mao.0000187238.56583.9b 16371853

[40] Fumiyuki Goto M Ken Hayashi M Takanobu Kunihiro M Kaoru Ogawa M. The possible contribution of angiitis to the onset of benign paroxysmal positional vertigo (BPPV). Int Tinnitus J (2010) 16(1):25–8. 21609909

[41] Tsai KL Wang CT Kuo CH Cheng YY Ma HI Hung CH The potential role of epigenetic modulations in BPPV maneuver exercises. Oncotarget (2016) 7(24):35522–34. 10.18632/oncotarget.9446 27203679 PMC5094942

[42] Maes M. Evidence for an immune response in major depression: a review and hypothesis. Prog Neuropsychopharmacol Biol Psychiatry (1995) 19(1):11–38. 10.1016/0278-5846(94)00101-M 7708925

[43] Maes M Berk M Goehler L Song C Anderson G Galecki P Depression and sickness behavior are Janus-faced responses to shared inflammatory pathways. BMC Med (2012) 10:66. 10.1186/1741-7015-10-66 22747645 PMC3391987

[44] Leonard B Maes M. Mechanistic explanations how cell-mediated immune activation, inflammation and oxidative and nitrosative stress pathways and their sequels and concomitants play a role in the pathophysiology of unipolar depression. Neurosci Biobehav Rev (2012) 36(2):764–85. 10.1016/j.neubiorev.2011.12.005 22197082

[45] Moylan S Maes M Wray NR Berk M. The neuroprogressive nature of major depressive disorder: pathways to disease evolution and resistance, and therapeutic implications. Mol Psychiatry (2013) 18(5):595–606. 10.1038/mp.2012.33 22525486

[46] Hall RC Hall RC Anxiety and endocrine disease. Semin Clin Neuropsychiatry (1999) 4(2):72–83. 10.1053/SCNP00400072 10378951

[47] Joyce PR. Neuroendocrine changes in depression. Aust N Z J Psychiatry (1985) 19(2):120–7. 10.3109/00048678509161309 2413836

[48] Chen ZJ Chang CH Hu LY Tu MS Lu T Chen PM Increased risk of benign paroxysmal positional vertigo in patients with anxiety disorders: a nationwide population-based retrospective cohort study. BMC Psychiatry (2016) 16:238. 10.1186/s12888-016-0950-2 27416989 PMC4946194

[49] Yetiser S Ince D. Demographic analysis of benign paroxysmal positional vertigo as a common public health problem. Ann Med Health Sci Res (2015) 5(1):50–3. 10.4103/2141-9248.149788 25745577 PMC4350063

[50] Ogun OA Buki B Cohn ES Janky KL Lundberg YW. Menopause and benign paroxysmal positional vertigo. Menopause (2014) 21(8):886–9. 10.1097/GME.0000000000000190 24496089 PMC4110114

[51] Vibert D Kompis M Häusler R. Benign paroxysmal positional vertigo in older women may be related to osteoporosis and osteopenia. Ann Otol Rhinol Laryngol (2003) 112(10):885–9. 10.1177/000348940311201010 14587980

[52] von Brevern M Seelig T Neuhauser H Lempert T. Benign paroxysmal positional vertigo predominantly affects the right labyrinth. J Neurol Neurosurg Psychiatry (2004) 75(10):1487–8. 10.1136/jnnp.2003.031500 15377705 PMC1738771

[53] Macias JD Lambert KM Massingale S Ellensohn A Ann Fritz J. Variables affecting treatment in benign paroxysmal positional vertigo. Laryngoscope (2000) 110(11):1921–4. 10.1097/00005537-200011000-00029 11081611

[54] Tan J Deng Y Zhang T Wang M. Clinical characteristics and treatment outcomes for benign paroxysmal positional vertigo comorbid with hypertension. Acta Otolaryngol (2017) 137(5):482–4. 10.1080/00016489.2016.1247985 27841099

[55] von Brevern M Seelig T Radtke A Tiel-Wilck K Neuhauser H Lempert T. Short-term efficacy of Epley’s manoeuvre: a double-blind randomised trial. J Neurol Neurosurg Psychiatry (2006) 77(8):980–2. 10.1136/jnnp.2005.085894 16549410 PMC2077628

[56] D’Silva LJ The Impact of Type 2 Diabetes Mellitus on Symptom Presentation and Response to Treatment in Individuals with Benign Paroxysmal Positional Vertigo. Lawrence, Kansas: University of Kansas (2016).

[57] Honaker JA Gilbert JM Shepard NT Blum DJ Staab JP. Adverse effects of health anxiety on management of a patient with benign paroxysmal positional vertigo, vestibular migraine and chronic subjective dizziness. Am J Otolaryngol (2013) 34(5):592–5. 10.1016/j.amjoto.2013.02.002 23578435

